# Concentration of 2,3 bisphosphoglycerate in cattle affected with acute ruminal acidosis

**DOI:** 10.1002/vms3.579

**Published:** 2021-07-17

**Authors:** Dhiyaa A. Moosa, Qaes T. Al‐Obaidi, Nashaat G. Mustafa, Mohammad O. Dahl

**Affiliations:** ^1^ Department of Internal and Preventive Medicine College of Veterinary Medicine University of Mosul Mosul Iraq; ^2^ Department of Physiology Biochemistry and Pharmacology College of Veterinary Medicine University of Mosul Mosul Iraq

**Keywords:** 2, 3‐BPG, ruminal pH, systemic acidosis, tissue hypoxia

## Abstract

The main objective of the study conducted here was to estimate the concentration of 2,3‐Bisphosphoglycerate (2,3‐BPG), 1,3‐Bisphosphoglycerate (1,3‐BPG), bisphospho‐glycerate mutase (BPGM) and 3‐phosphoglycerate (3PG) in cattle clinically diagnosed with acute ruminal acidosis. A secondary objective was to examine the physical and chemical characteristics of the ruminal fluid in affected cattle. A total of 20 cattle clinically diagnosed with acute ruminal acidosis and eight clinically normal cattle were included in this study. The results showed that decrease of ruminal pH changed the ruminal fluid colour, odour and consistency, as well as decreased the sedimentation time, increased the methylene blue reduction time, and decreased ruminal microflora motility. The study indicated that the concentration of 2,3‐BPG, BPGM and BPGP decreased with the decrease of ruminal pH, while 3‐PG concentration was not affected with the decrease of ruminal pH. In conclusion, 2,3‐BPG could play a role in the pathogenesis of ruminal acidosis, and thus, the intravenous administration of sodium bicarbonate is important, particularly in severe cases, to correct any systemic acidosis that can decrease 2,3‐BPG concentration and results in tissue hypoxia.

## Graphical abstract

Concentration of 2,3‐BPG in cattle diagnosed as acute ruminal acidosis was estimated under a field condition. The concentration of 2,3‐BPG, BPGM, and BPGP decreased with decrease in ruminal pH.

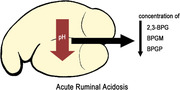

## 1 INTRODUCTION

2,3‐Bisphosphoglycerate (2,3‐BPG) is an important acid for the efficiency of haemoglobin function as an oxygen transporter (Berg et al., 2007). About 35% of the variation in the affinity of haemoglobin to oxygen is due to the change in the concentration of 2,3‐BPG (MacDonald, 1977). For instance, haemoglobin affinity for the oxygen is decreased due to binding of 2,3‐BPG to the haemoglobin which promotes release of oxygen from erythrocytes to tissues (Lieberman & Peet, 2015). 2,3‐BPG, also known as 2,3‐diphosphoglycerate (2,3‐DPG), is formed from 1,3‐Bisphosphoglycerate (1,3‐BPG) by the action of bisphosphoglycerate mutase (BPGM) and broken down to 3‐phosphoglycerate (3PG) as a function of bisphosphoglycerate phosphatase (BPGP) activity (Kaneko et al., 2008).

Different conditions can be associated with the change in 2,3‐BPG concentration. For instance, a study included 18 adult sheep revealed that the administration of thyroxine increased the concentration of 2,3‐BPG as a function of direct effect on the BPGM enzyme (Kaneko et al., 2008; Studzinski et al., 1982). In ruminants and cats, however, the concertation of 2,3‐BPG is relatively low due to low BPGM activity, although cattle have moderate BPGM activity, in contrast to that for horses and dogs as well as human that have high 2,3‐BPG concentration (Harvey, 2012; Kaneko et al., 2008). In human, on the other hand, decrease in 2,3‐BPG concentration has been found be associated with acidosis (Ibrahim et al., 2005). It is not known if ruminant affected with acute ruminal acidosis and systemic acidosis can show decrease in 2,3‐BPG.

Acute ruminal acidosis is a common digestive disorder in ruminants. It is usually results from consumption of large quantities of highly digestible or carbohydrate‐rich ration, which can alter the ruminal microbial inhabitants to those that produce more lactic acid, and thus decrease the ruminal pH below 5 leading to different pathophysiological consequences such as mycotic rumenitis, laminitis and metabolic acidosis (Constable et al., 2017). Consequently, metabolic acidosis can result from absorption of lactic acid and other organic acids from the rumen, reduction of plasma bicarbonate reserves and dehydration (Howard, 1981).

In a local study, gastrointestinal disturbances, including acute ruminal acidosis, were the most common cases received at a veterinary teaching hospital (Dahl et al., 2021). Thereupon, it is important to add details to the pathogenesis of such disturbances, as these conditions are considered the most frequent cases that can a veterinarian see in the career (Belknap & Navarre, 2000). The hypothesis of the current study was that ruminal acidosis leads to decrease the concentration of 2,3‐BPG as a consequence to decrease blood pH, which can result in tissue hypoxia and exacerbate the impact of ruminal acidosis on the affected animal. To our knowledge, no study has examined the concentration of 2,3‐BPG in cattle affected with acute ruminal acidosis. Therefore, the main objective of the study conducted here was to estimate the concentration of 2,3‐BPG, 1,3‐BPG, BPGM and 3PG in cattle clinically diagnosed with acute ruminal acidosis. A secondary objective was to examine the physical and chemical characteristics of the ruminal fluid in affected cattle.

## 2 MATERIALS AND METHODS

### 2.1 Study animals

A total of 20 local‐breed cattle clinically diagnosed with acute ruminal acidosis and eight clinically normal cattle were included in this study. A convenience sample was considered for this study as it can be used in such analytical studies (Dohoo et al., 2003), and because of fund limitations. A routine clinical examination was performed on all the studied cattle.

### 2.2 Ruminal fluid collection and examination

Ruminal fluid was collected using a locally manufactured device consisting of a short hose attached to a suction pump as previously described (Klopp et al., 2018). The physical and laboratory examination were immediately performed to the samples in order to avoid alteration in the ruminal fluid properties as a result of exposure to the air. The following characteristics for the ruminal fluid were examined: colour, odour, consistency, pH, sedimentation activity, methylene blue reduction time and ruminal microflora motility (Freeman & Klenner, 2015), taking into the consideration that methylene blue reduction time reflects the activity of the ruminal microflora, as active microflora can decolorizes the dye within 3–6 min (Bayne & Edmondson, 2021).

### 2.3 Estimation of 2,3‐BPG and its related enzymes and acid

A total of approximately 5 ml of the blood was gently collected from the jugular vein using a syringe with 18G needle (Green Rose, China) and placed in an EDTA tube (AFCO, Jordon) for plasma collection. Plasma was used for the estimation of bovine 2,3‐BPG and its related enzymes including BPGM and BPGP, as well as the 3‐PG. For this purpose, ELISA technique was conducted using commercial kits (MBS, Canada).

### 2.4 Statistical analysis

Linear regression was used to study the relationship between the decrease of ruminal pH and the change in ruminal contraction, ruminal fluid sedimentation activity, methylene blue reaction, ruminal microflora motility, as well as plasma concentration of 2,3‐BPG, BPGM, BPGP and 3‐PG. Additionally, animals diagnosed with acute carbohydrate engorgement were divided into three groups on a basis of ruminal fluid pH as the following: (i) animals with approximately normal rumen (pH ≥ 6); (ii) animals with moderate degree of ruminal abnormality (pH = 5); and (iii) animals with severe grain overload (pH < 5) (Constable et al., 2017). One way ANOVA was used to compare ruminal contraction, ruminal fluid sedimentation activity, methylene blue reduction time, as well as plasma concentration of 2,3‐BPG, BPGM, BPGP and 3‐PG between designated groups as well as normal cattle group (Moore et al., 2009). Multiple pairwise comparisons between study groups were achieved using Bonferroni correction (Armstrong, 2014). In all analyses, a value of *Pp* ≤ 0.05 (two‐tailed) was considered significant. The statistical analyses were performed using STATA 13.0 (StataCorp, College Station, TX).

## 3 RESULTS

### 3.1 Ruminal fluid

**FIGURE 1:**
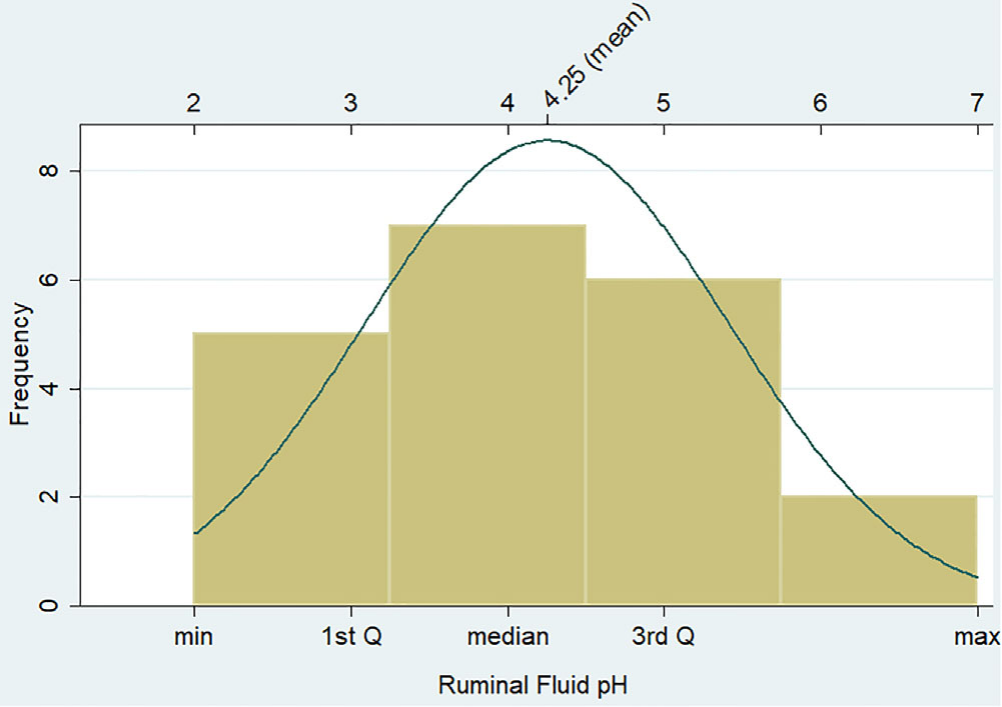
Descriptive statistics of the ruminal fluid pH in the cattle clinically diagnosed with acute ruminal acidosis

**FIGURE 2:**
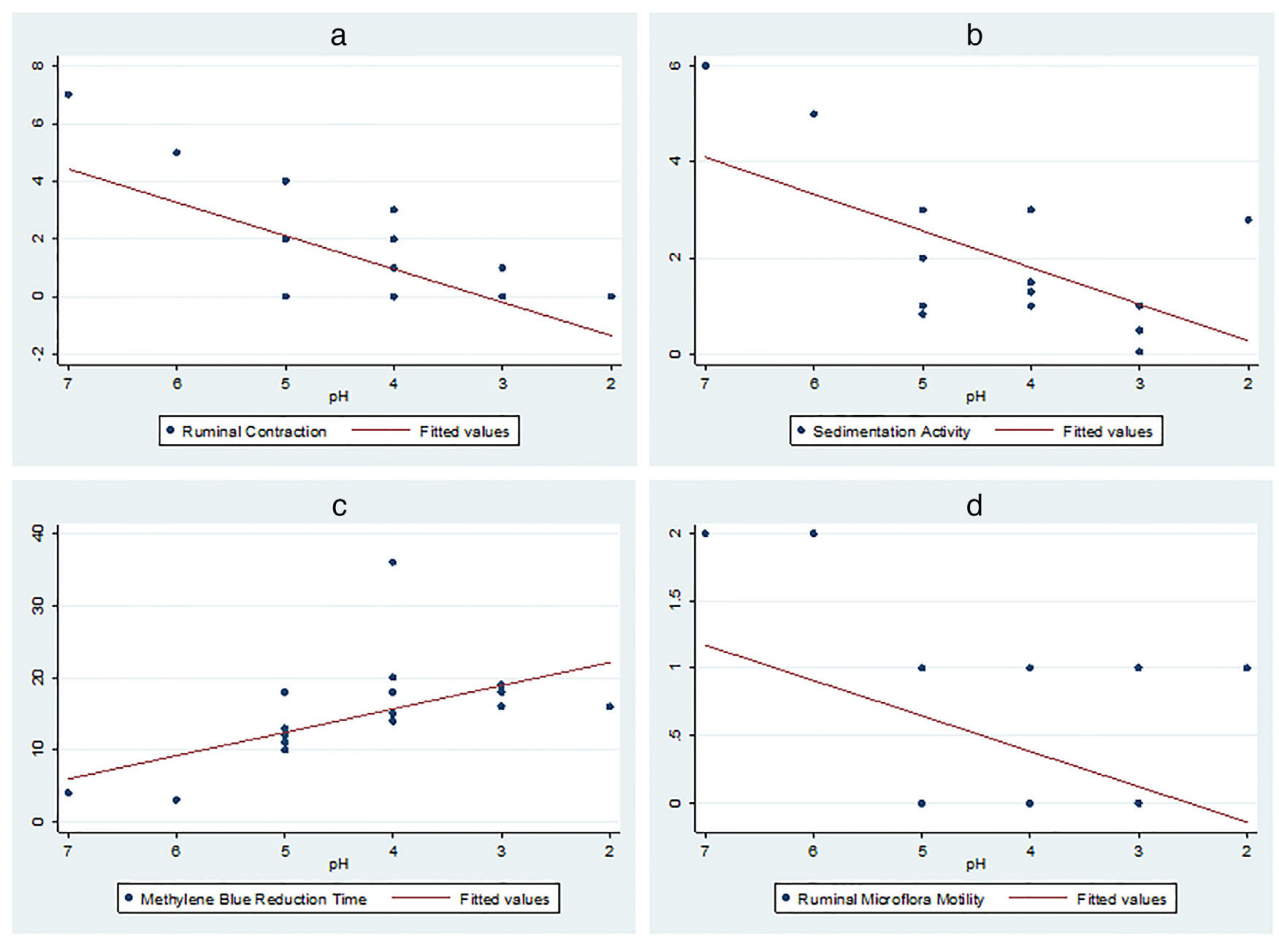
Ruminal contraction and ruminal fluid characteristics as a function of changes in ruminal pH in cattle clinically diagnosed with acute ruminal acidosis

Mean and median of ruminal fluid pH in the cattle clinically diagnosed with acute ruminal acidosis were 4.25 and 4, respectively (Figure 1). The colour of the ruminal fluid for the majority of those cattle was milky grey, and some had yellowish brown or greenish black. The odour and consistency of the ruminal fluid were watery and sour, respectively, except those with pH of 6 and 7 (*n* = 2) which were aromatic and viscus. Clinically, ruminal contraction decreased by 1.2 times for each unit decrease in ruminal pH (*Pp* = 0.006; Figure 2a). On the other hand, ruminal fluid sedimentation time was decreased by 0.76 min for each unit decrease in ruminal pH (*Pp* = 0.05; Figure 2b), while methylene blue reduction time increased by 3.2 min for each unit decrease in ruminal pH (*Pp* = 0.001; Figure 2c). In addition, although ruminal microflora motility decreased when ruminal pH decreased (Figure 2d), the linear coefficient did not reach the statistical significance level (*Pp* = 0.15). Finally, ruminal contraction and ruminal sedimentation activity significantly decreased in cattle with moderate and severe cases compared to those about normal cattle, while the methylene blue reduction time significantly decreased as the severity of the case increased (*Pp* < 0.05, Table 1).

**TABLE 1:** Ruminal contraction and ruminal fluid characteristics changes (mean ± standard deviation) in cattle clinically diagnosed with acute ruminal acidosis compared to those normal cattle

		Cattle with acute ruminal acidosis		
Parameter	Normal cattle	About normal pH ≥ 6	Moderate pH = 5	Severe pH < 5
Ruminal contraction per 5 minutes	8 ± 0.8a	6 ± 1.4a	1 ± 1.7b	0.6 ± 1.0b
Sedimentation activity (minutes)	6 ± 0.5a	5.5 ± 0.7a	1.6 ± 0.8b	1.6 ± 1.1b
Methylene blue reduction time (minutes)	3 ± 0.2a	3.5 ± 0.7a	12.3 ± 3.0b	18 ± 6.0c

Different letters (i.e., a, b, c) refer to statistical difference (*Pp* ≤ 0.05).

### 3.2 Concentrations of 2,3‐BPG, 1,3‐BPG, BPGM and 3PG

**FIGURE 3:**
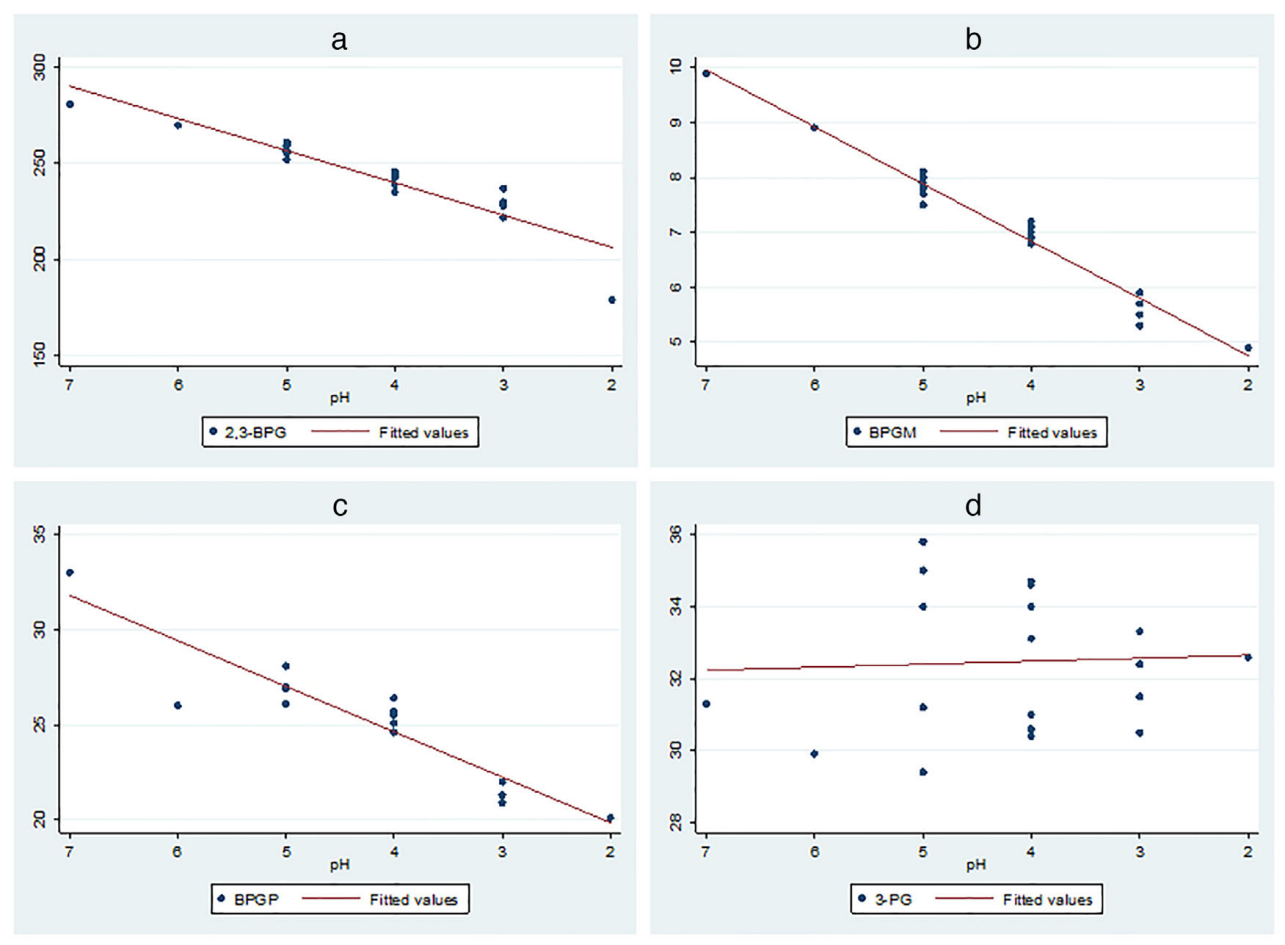
Concentration of 2,3‐BPG, 1,3‐BPG, BPGM and 3PG in the plasma as a function of changes in ruminal pH in cattle clinically diagnosed with acute ruminal acidosis

The analysis indicated that the concentrations of 2,3‐BPG, BPGM and BPGP in plasma decreased by 16.8 IU/g, 1.04 IU/g and 2.4 IU/mg, respectively, for each unit decrease in ruminal pH (*Pp* < 0.05; Figure 3a–c). In contrast, the concentration of 3‐PG increased by 0.08 IU/g for each unit decrease in ruminal pH; however, the linear coefficient did not reach the statistical significance level (*Pp* = 0.79). The concentration of 2,3‐BPG and BPGP significantly decreased in cattle with severe cases compared to those with moderate and about normal cases as well as normal cattle (*Pp* < 0.05), BPGM decreased as the severity of the cases increased (*Pp* < 0.05), while the concentration of 3‐PG did not affected by the severity of the cases (Table 2).

**TABLE 2:** Concentrations of 2,3‐BPG, 1,3‐BPG, BPGM and 3PG in the plasma (mean ± standard deviation) in cattle clinically diagnosed with acute ruminal acidosis compared to those normal cattle

		Cattle with acute ruminal acidosis		
Parameter	Normal cattle	About normal pH ≥ 6	Moderate pH = 5	Severe pH < 5
2,3‐BPG (IU/g)	290.5 ± 14.6a	275.5 ± 7.8a	257.5 ± 3.3a	232.3 ± 18.3b
BPGM (IU/g)	10.3 ± 2.4a	9.4 ± 0.7a	7.8 ± 0.2b	6.4 ± 0.8c
BPGP (IU/mg)	30.1 ± 4.6a	29.5 ± 4.9a	27.0 ± 0.7a	23.6 ± 2.4c
3‐PG (IU/g)	32.3 ± 4.4a	30.6 ± 1.0a	33.2 ± 2.4a	32.4 ± 1.6a

Different letters (i.e., a, b, c) refer to statistical difference (*Pp* ≤ 0.05).

## 4 DISCUSSION

The study conducted here was an attempt to add new information concerning the pathogenesis of acute ruminal acidosis in ruminants, a frequent case a veterinarian can face. It is the first study that examined the effect of ruminal acidosis on the concentration of 2,3‐BPG, an important intra‐erythrocyte compound for haemoglobin function. The study indicated that the concentration of 2,3‐BPG decreased when the rumen pH became 5 or less. Although current study included a convenience sample, the power was 100% (OpenEpi; https://www.openepi.com/Power/PowerMean.htm).

### 4.1 Ruminal fluid

The current study confirmed what was previously known that the decrease of ruminal pH had significant impact on the ruminal fluid colour, odour, consistency, sedimentation activity, methylene blue reduction time and microflora motility. The colour, odour, and consistency of ruminal fluid in study cattle were in‐line with what has been indicated to be found in ruminants with high grain feeding and affected with ruminal acidosis (Ivany et al., 2002). In addition, the decrease in the sedimentation time and increase in the methylene blue reduction time in the study cattle indicated an inactive microflora leading to impairment of ruminal contraction (Constable et al., 2017), which was confirmed by the cease of ruminal microflora activity. The analysis did not show statistical significance in the decrease in the microflora activity with increase of the case severity because the motility became zero when rumen pH decreased to 5 and lower. All these changes are attributed to the direct effect of low pH on the rumen ecosystem (Jaramillo‐López et al., 2017).

### 4.2 Concentrations of 2,3‐BPG, 1,3‐BPG, BPGM and 3PG

In this study, the concentration of 2,3‐BPG, BPGM and BPGP decreased with the decrease in ruminal pH. However, the concentration of 2,3‐BPG significantly decreased when the ruminal pH became lower than 5. An interpretation to the decrease in 2,3‐BPG and its related enzymes is that a metabolic acidosis was developed as a function of crossing the lactic acid to the blood stream through ruminal wall, as well as a function of dehydration (Hernandez et al., 2014; Ivany et al., 2002). However, blood pH was not estimated in study cows, which is a limitation. It has been indicated that the activity of BPGM in the erythrocytes of cattle is moderately low (Kaneko et al., 2008), which might explain why 2,3‐BPG did not significantly decreased with moderate cases of acidosis, while BPGM decreased. Finally, more studies are needed to confirm the results of the current study.

## 5 CONCLUSIONS

Although the pathogenesis of ruminal acidosis is well studied, 2,3‐BPG could play a role in its pathogenesis. That is, the decrease in oxygen supply to peripheral tissues happened as a sequel of lactic acidosis in ruminal acidosis cases might be attributed to decrease in 2,3‐BPG concentration. Therefore, an intravenous administration of sodium bicarbonate is important, particularly in severe cases, to correct any systemic acidosis that can decrease 2,3‐BPG concentration and results in tissue hypoxia.

## ETHICAL STATEMENT

The authors confirm that the ethical policies of the journal, as noted on the journal's author guidelines page, have been adhered to. No ethical approval was required as this article is based on field work not involving any experiment.

## CONFLICT OF INTEREST

The authors declare that there is no conflict of interest in the research.

## AUTHOR CONTRIBUTIONS

D.A. Moosa and Q.T. Al‐Obaidi: sampling and laboratory examination. N.G. Mustafa: laboratory examination. M.O. Dahl: data curation, formal analysis, writing the final draft. All authors: conceptualization and methodology.

### PEER REVIEW

The peer review history for this article is available at https://publons.com/publon/10.1002/vms3.579

## Data Availability

The data that support the findings of this study are available from the corresponding author upon reasonable request.
